# Chemical speciation and fate of tripolyphosphate after application to a calcareous soil

**DOI:** 10.1186/s12932-017-0046-z

**Published:** 2018-01-08

**Authors:** Jordan G. Hamilton, Jay Grosskleg, David Hilger, Kris Bradshaw, Trevor Carlson, Steven D. Siciliano, Derek Peak

**Affiliations:** 1 Department of Soil Science, University of Saskatchewan, Saskatoon, SK S7N 5A8 Canada; 2 Federated Cooperatives Ltd., Saskatoon, SK S7K 0H2 Canada; 3 Department of Earth and Environmental Sciences, University of Waterloo, Waterloo, ON Canada; 4 Geosyntec Consultants, Saskatoon, SK S7P 0A4 Canada

**Keywords:** Tripolyphosphate adsorption, Phosphorus amendment, Phosphorus XANES, Calcium phosphate minerals

## Abstract

Adsorption and precipitation reactions often dictate the availability of phosphorus in soil environments. Tripolyphosphate (TPP) is considered a form of slow release P fertilizer in P limited soils, however, investigations of the chemical fate of TPP in soils are limited. It has been proposed that TPP rapidly hydrolyzes in the soil solution before adsorbing or precipitating with soil surfaces, but in model systems, TPP also adsorbs rapidly onto mineral surfaces. To study the adsorption behavior of TPP in calcareous soils, a short-term (48 h) TPP spike was performed under laboratory conditions. To determine the fate of TPP under field conditions, two different liquid TPP amendments were applied to a P limited subsurface field site via an in-ground injection system. Phosphorus speciation was assessed using X-ray absorption spectroscopy, total and labile extractable P, and X-ray diffraction. Adsorption of TPP to soil mineral surfaces was rapid (< 48 h) and persisted without fully hydrolyzing to ortho-P. Linear combination fitting of XAS data indicated that the distribution of adsorbed P was highest (~ 30–40%) throughout the site after the first TPP amendment application (high water volume and low TPP concentrations). In contrast, lower water volumes with more concentrated TPP resulted in lower relative fractions of adsorbed P (15–25%), but a significant increase in total P concentrations (~ 3000 mg P kg soil) and adsorbed P (60%) directly adjacent to the injection system. This demonstrates that TPP application increases the adsorbed P fraction of calcareous soils through rapid adsorption reactions with soil mineral surfaces.

**Electronic supplementary material:**

The online version of this article (10.1186/s12932-017-0046-z) contains supplementary material, which is available to authorized users.

## Introduction

Tripolyphosphates (TPP) have been commonly used as a phosphorus (P) source in slow release liquid fertilizers [1–3]. To be bioavailable to plant or microbial communities, TPP must first be hydrolyzed to phosphate monomers (ortho-P). Tripolyphosphate is believed to persist in the soil solution until undergoing hydrolysis, when it becomes bioavailable and reactive in the soil environment [4–6]. However, there is significant evidence that suggests TPP and other linear polyphosphates adsorb directly to metal oxide surfaces without having to first be hydrolyzed [7–11]. If TPP adsorbs directly to soil mineral surfaces, this could not only reduce TPP mobility in the soil solution but also reduce calcium phosphate (Ca-P) mineral precipitation. Calcium phosphate mineral formation immobilizes P from the soil solution, reducing the fraction of readily bioavailable P.

Tripolyphosphate or linear polyphosphate applications to calcareous soils may be a novel way to improve P nutrient availability. Since linear polyphosphates must undergo hydrolysis (either biotic or abiotic) to ortho-P before precipitating as a mineral phase with either Ca or Fe (pH dependent), they can act as a slow release fertilizer [7]. In the soil environment TPP hydrolysis can often be biotically catalyzed by the phosphatase enzyme excreted from plants as root exudates or by microbes [12–14]. In a healthy soil environment, TPP has been thought to rapidly hydrolyze due to an abundance of exogenous phosphatase in the soil solution exuded to mobilize organic P [15]. However, this relies upon an active soil biological pool, as phosphatase only persists for a few days in a non-sterile environment [12, 14]. Research has found that polyphosphate adsorption to mineral surfaces likely reduces enzyme catalyzed hydrolysis [16, 17]. In the absence of rapid hydrolysis by phosphatase, abiotic factors will play a role in hydrolyzing TPP, however at significantly slower rates.

Under cool, alkaline environmental conditions, abiotic hydrolysis rates of TPP are slow as both temperature and pH strongly affect this process [3, 7, 18]. For example, at temperatures below 25 °C, under sterile solution conditions, hydrolysis of TPP completely stalls, whereas at temperatures above ~ 50 °C the hydrolysis of TPP is rapid [3]. Both McBeath et al. [3] and Zinder et al. [18] found that solution pH has an inverse relationship with TPP hydrolysis. The half-life of TPP at pH 2.3 was 34 days while at pH 5.4 it was found to be 174 days. Both papers hypothesized that soluble cations in solution can catalyze TPP hydrolysis. Tripolyphosphates are also capable of adsorbing directly to mineral oxide surfaces without first hydrolyzing to ortho-P [8, 10]. Researchers have also shown [7] that the adsorption of TPP to mineral surfaces can catalyze the hydrolysis of TPP to pyrophosphate (pyro-P) and ortho-P. This provides evidence that TPP adsorption onto mineral surfaces is likely to play an important role in hydrolysis and thus chemical fate of TPP in soils.

Phosphate (${\text{PO}}_{4}^{3 - }$) rapidly forms both adsorption complexes and precipitate phases which can limit P availability. The speciation and chemical fate of P is directly dependent on the soil solution and geochemical conditions. At acidic pH, ortho-P adsorbs and forms surface precipitates on Al-oxides (i.e., berlinite, and variscite) and Fe(III) oxide (i.e. strengite) mineral surfaces [19, 20]. The formation of these precipitates removes P from the soil solution and decreases the overall bioavailability of P [20]. At alkaline pH and in calcareous systems, ortho-P forms a variety of calcium phosphate (Ca–P) phases with the solubility-limiting phases depending upon several factors including: pH, Ca:P ratio, and the presence of competing ions in solution such as ${\text{NH}}_{4}^{ + }$ and Mg^2+^ [20–23]. The presence of ${\text{NH}}_{4}^{ + }$ and Mg^2+^ can lead to the formation of more soluble phosphate minerals such as struvite (NH_4_MgPO_4_·6H_2_O), amorphous calcium phosphate (ACP) and dicalcium phosphate (brushite) [24, 25]. The formation of ACP, brushite, and hydroxyapatite is also largely dependent on Ca:Mg:P ratios [22, 23]. Higher Ca:P ratios favour the formation of crystalline and less soluble phases like hydroxyapatite [22, 23], whereas the incorporation of even small amounts of Mg into the crystal structure of Ca-P minerals can poison the growth sites and prevent the formation/transition to hydroxyapatite [21].

Several spectroscopic techniques are available to study P speciation in soils and geochemical systems. The most commonly used X-ray technique for determining P speciation in soils is X-ray absorption near edge structure (XANES) spectroscopy which is sensitive to the average local bonding environment of P atoms [19, 24, 26]. A XANES spectrum of any sample is a weighted average of all P atoms measured, which has the potential to overlook minor species that contribute less scattering to the spectrum [24]. One can use reference spectra and linear combination fitting (LCF) to estimate P-species [19, 27–31]. However, LCF has the risk of over-estimating the spectral contributions from P species with atoms that strongly scatter X-rays (i.e. Ca) in Ca-P minerals whereas species that contribute minimal structure (adsorbed P) may be underrepresented [24, 28]. This issue is compounded at the P K-edge due to overlapping spectral features of many P species. For example, the challenges of determining the different types of TPP, pyro-P, and ortho-P adsorption complexes with XANES spectroscopy is highlighted by Hamilton and coworkers [7] where adsorbed TPP on goethite is spectrally identical to adsorbed pyro-P and adsorbed ortho-P. Unfortunately, the complex nature of soils and the combination of P species (adsorbed/mineral phases) present prevents the direct measurement of soil adsorbed TPP by techniques more suitable to identification of polyphosphates, namely Fourier Transform Infrared or Nuclear Magnetic Resonance spectroscopic methods [19]. Nonetheless, our recent P K-edge XANES study of a model system allows us to infer the speciation of adsorbed TPP based upon the known adsorption and precipitation mechanisms on a goethite surface in the presence of Ca^2+^ [7].

The objectives of this study were (a) to determine the short-term chemical fate of TPP in soils and (b) to characterize the long-term fate and mobility of two TPP nutrient applications applied to a P-limited calcareous soil. To study the adsorption potential of TPP to soil minerals and the effect this has on mobility, TPP was applied to a P limited subsurface soil under short-term lab conditions and to a P limited field site to track the chemical fate of TPP under longer-term environmental conditions. The effectiveness of TPP as a P amendment will be gauged upon whether TPP adsorbs directly to soil mineral surfaces or whether ortho-P precipitation reactions dominate. The goals of this study are (1) to determine whether TPP will adsorb directly to soil mineral surfaces under short-term reaction conditions and (2) to determine the chemical fate and mobility of two TPP amendment applications to a calcareous P limited subsurface soil system.

## Materials and methods

### Site history and soil sampling

**Fig. 1:**
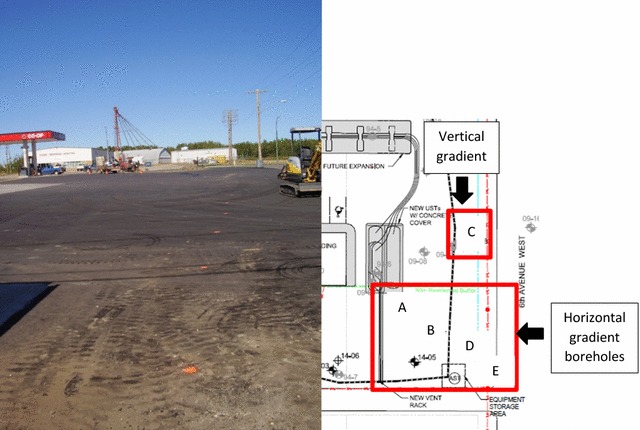
Site picture (Left) with location of the injection line indicated by orange dashed line. Schematic/site map (Right) of Meadow Lake owned and operated Federated Coop fueling station. Dashed (black) line indicates the amendment injection line used for nutrient application. The vertical borehole (C) for measuring TPP infiltration through the soil profile is located directly adjacent to the injection line, while the horizontal boreholes (A, B, D, and E) are 3 and 6 m from the injection system. Location A corresponds to site labels 1–2, B: 3–4, C: 5–7, D: 8–9, and E: 10–11

The study site is a Federated Cooperatives Ltd (FCL) owned and operated fueling station that also historically served as a fertilizer storage facility. The onsite fueling station currently consists of a 4 pump/8 line gas bar with underground storage tanks (see Fig. 1 for the site and sampling schematic). Petroleum hydrocarbon contamination (PHC) originated from leaking bulk storage tanks, which have been replaced as part of an upgrade to the current residential fueling station. Groundwater is routinely monitored throughout the site to track the extent of hydrocarbon movement and nutrient concentrations. This site was chosen for TPP application because it is part of an active in situ bioremediation study and has been identified as being highly P limited, determined through P groundwater concentrations of < 0.3 mg P/L. This groundwater monitoring has identified that the PHC is not moving offsite.

Tripolyphosphate nutrient amendments were applied through two underground perforated injection lines that were installed as part of a gravity fed amendment delivery system. The injection lines are at a depth of 1.22 m and they rely upon preferential flow paths to transport the nutrient solution to the hydrocarbon contaminated soil zone between 1.82 and 3.66 m. The first amendment application was performed prior to our involvement as part of an in situ bioremediation trial to improve nutrient conditions throughout the site; this first nutrient application consisted of urea (9.5 kg) and sodium tripolyphosphate (1.4 kg) diluted in 13,500 L of water. It was noted during this application that the study area of the site had initially become saturated with higher water volumes then the infiltration capacity of the site, resulting in some mounding of the site’s groundwater. One year after the TPP application, soil cores (Fig. 1) were collected directly adjacent to the injection line as well as up and down-gradient of the main injection line. After the first amendment application, no groundwater P was detected. A second amendment application occurred 3 years after the first amendment, consisting of a larger TPP (102 kg) and urea N (9.5 kg) amendment spike diluted in 4500 L of water. A second set of sample cores were collected 1 year later along the same gradient illustrated in Fig. 1.

Soils were sampled via coring using a push drill rig collecting 2″ diameter soil cores to a depth of 4.26 m. The cores were immediately sealed, transported on ice, and frozen before subsampling to limit potential oxidation effects on soil mineralogy. Soil cores were subsampled by collecting ~ 30 g from each of the studied depths. These subsamples were freeze dried, ground, and homogenized for elemental and spectroscopic analysis. Analysis of the soil cores focused on the 1.82 and 3.66 m depths. The rationale for choosing these depths was that the 1.82 m depth is close but below the amendment injection system, whereas the 3.66 m depth is a sand lens that represents the leading edge of the hydrocarbon plume.

### Short-term adsorption of TPP

Two soils (1.82 and 3.66 m) from the research site were used to determine the short-term sorption potential of TPP with soil minerals. The soils were suspended in 0.01 M NaCl background electrolyte solution and adjusted to pH 6.5 using 0.01 M H_2_SO_4_. All soil treatments were spiked (using either TPP or ortho-P) to a targeted loading of 10,000 mg P/kg of soil. The ortho-P source was K_2_HPO_4_ and TPP was applied as Na-TPP; both in double deionized water. After P addition, the pH was adjusted as needed over 48 h to maintain pH 6.5. The soils were then filtered through a 0.45 µm filter paper and triple washed with background electrolyte to remove entrained P. Reacted soil samples were freeze-dried and ground for XAS analysis to determine complexation mechanisms.

### XAS and XRD Data collection and analysis

X-ray absorption spectroscopic (XAS) and X-ray diffraction (XRD) measurements were conducted at the Canadian Light Source (CLS) synchrotron in Saskatoon, SK., Canada. The Canadian Light Source operates a storage ring at 2.9 GeV and between 150 and 250 mA. All P K-edge XANES measurements were collected at the SXRMB beamline (06B1-1) utilizing an InSb (111) monochromator in fluorescence mode under vacuum conditions with a 4-element Vortex detector. Concentrated reference standards were diluted with boron nitride to ~ 1 wt. % total P to minimize self-absorption effects. Soil samples were dried, ground to a uniform particle with mortar and pestle, and applied to the beamline sample holder as a thin layer on carbon tape. The beam spot size was 1 × 3 mm giving a bulk representation of the P speciation of each soil sample. See supplemental information for the preparation conditions for adsorption standards. The Ca and Mg phosphate mineral reference standards were synthesized by Hilger [32]. All other compounds were purchased and were reagent grade or better.

All P XANES spectra were processed and linear combination fit (LCF) using the DEMETER software package [33]. Briefly, data was processed with background removal, calibration to an internal reference standard, alignment and then merging of scans. Phosphorus reference spectra used in the LCF model fits are located in the (Additional file 1: Figure S1). It is known that there is an inherent level in uncertainty in LCF of unknown XANES spectra typically estimated at ± 10% or less [28, 30]. To reduce the uncertainty and reliance on the statistical output of the LCF model results, all available geochemical information was incorporated in selecting the reported LCF model. These conditions included soil pH, total and labile P concentrations, soil mineralogy, as well as groundwater Ca and Mg concentrations. The statistical based nature of LCF has difficulty distinguishing between reference compounds that have similar structure such as calcium phosphate mineral species. The LCF results for all Ca-P mineral phases were reported as a single summed value for two reasons [1] due to DEMETER fitting multiple reference compounds to the same spectral features, and [2] limited data quality, due to low concentrations of P in these soils which limited data quality and was a concern that it may potentially increase LCF uncertainty; specifically with fitting multiple mineral phases with similar spectral features.

Linear combination fitting was performed with only one adsorbed P standard due to the similarities and lack of identifying spectral features between the “adsorbed ortho-P” and “adsorbed TPP” reference spectra. It was determined throughout the LCF analysis that either adsorbed P reference spectra would provide an identical model fit result. The adsorbed P fraction of the LCF model fits are operationally defined as adsorbed TPP. This operational definition is based upon several factors: [1] adsorbed TPP is indistinguishable from adsorbed ortho-P (Additional file 1: Figure S1) [2]. In the presence of high Ca concentrations ortho-P would rapidly precipitate and not persist as adsorbed P in a calcareous soil environment. Groundwater modeling of the system has indicated that even low concentrations of groundwater ortho-P would be oversaturation with respect to calcium phosphate mineral precipitation, and as such adsorbed ortho-P is not expected to a be present as a phase [3]. Tripolyphosphate adsorbs directly to mineral surfaces without first hydrolyzing to ortho-P [7, 8, 10]. Tripolyphosphate has been shown under lab conditions to remain adsorbed to mineral surfaces without hydrolyzing for several months at pH 8.5 [7]. Tripolyphosphate hydrolysis in cold climates and slightly alkaline soils (temp. < 5 °C) could potentially take several years to naturally occur given limited microbial activity; however, surface-catalyzed hydrolysis may be an important mechanism resulting in adsorbed TPP hydrolysis [3, 7, 18].

X-ray diffraction measurements were completed at the CMCF-BM (08B1-1) beamline utilizing an energy of 18 keV and a wavelength of 0.6888 Å. The beamline employs a Rayonix MX300-HE wide area detector to collected XRD data over a range of 2 –37  2θ (Å). Soils were ground to a uniform particle size with mortar and pestle and then loaded into a polyimide tube for analysis. Data processing was completed with the GSAS-II software package [34]. Phase identification of all XRD spectra was completed with X’Pert HighScore Plus (PANAnalytical) with Rietveld refinements completed using the GSAS and EXPGUI software package [35]. All crystallographic information used during the Rietveld refinements were taken from the mineral phases identified with X’Pert HighScore Plus.

### Soil extractions and analysis

Total elemental concentrations of all samples were determined with X-ray fluorescence (XRF) using ThermoFisher Scientific ARL OPTIM’X X-ray Analyzer. Dried soil samples were ground to a uniform particle size with mortar and pestle for XRF analysis. Elemental concentrations were determined using the OPTIQUANT software package which provides a ± 10% accuracy on converting counts per second into mg/kg elemental concentrations. X-ray fluorescence elemental analysis was selected because it is a non-destructive technique, while a single measurement provides the elemental concentrations of all the elements within each sample. Phosphorus concentrations were verified for accuracy by microwave soil digestions (US EPA Method 3051) with P concentrations measured using the colourmetric (molybdenum blue) method with a SEAL Analytical Inc. AutoAnalyzer 1 (AA1). Labile P fraction was operationally defined as the sum of P extracted from the sequential extraction steps of double deionized H_2_O (DDI) and 0.5 M Na-bicarbonate solution [36]. The extraction procedure consisted of a soil:solution ratio of 1:80 (w/v) for each sequential extraction step with the supernatant being filtered through a 0.45 µm filter and analyzed for P with an AutoAnalyzer 1. Soil pH was determined using a 0.01 M CaCl_2_ solution and a soil to solution ratio of 1:10 (w/v) [37–39]. The soil-solution slurry was mixed via end over end shaking for 0.5 h and then left to settle for 2 h before pH measurement.

## Results and discussion

### Short-term TPP adsorption

**Fig. 2:**
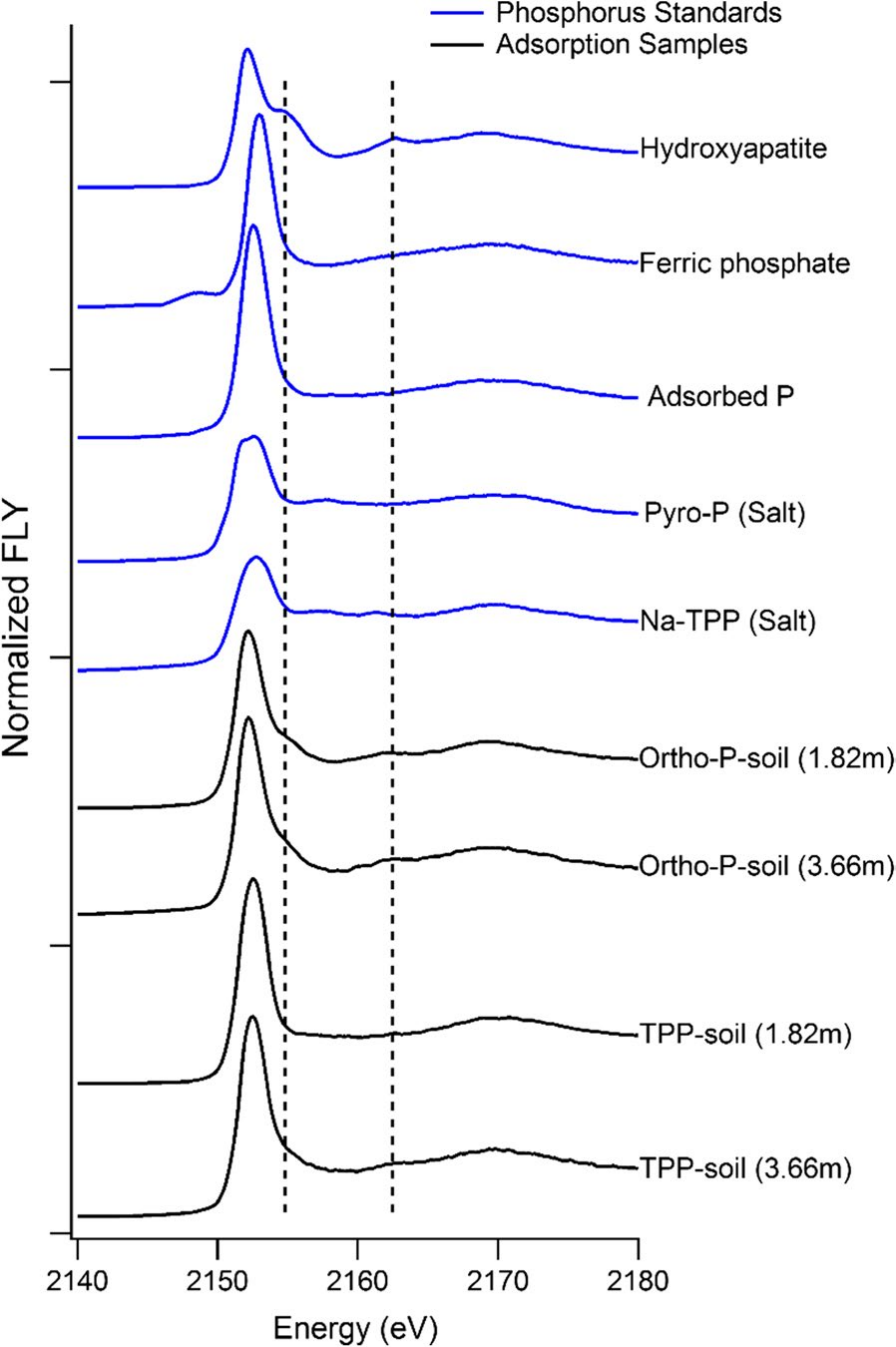
Phosphorus XANES spectra of the short-term (48 h) reaction of TPP and ortho-P with calcareous soils from two (1.82 and 3.66 m) depths of the study site

A number of researchers have shown that TPP rapidly adsorbs to metal oxide surfaces [7–11] but the mechanism of TPP sorption onto soils has not been previously determined. Our experimental results demonstrate (Fig. 2) that TPP directly adsorb to our study soils without first hydrolyzing to ortho-P. The P XANES indicate that, after 48 h of reaction, TPP has formed an adsorption complex consistent with the adsorbed TPP reference standard. In contrast, the XANES features of the 48 h ortho-P treatment show that ortho-P precipitated as a Ca-P phase based upon diagnostic spectral features (noted by dashed lines). This strongly suggests that TPP can adsorb directly to soils without first hydrolyzing to ortho-P in the soil solution; if hydrolysis occurred in solution then Ca-P precipitates would also form in the TPP samples. It is possible that adsorbed TPP will slowly hydrolyze on these mineral surfaces with the hydrolysis rates dependent on enzyme activity and geochemical conditions [3, 7, 18]. The 3.66 m TPP spiked soil does contain slight spectral features associated with the presence of Ca-P minerals species, but this is likely due to lower TPP adsorption to this sandy soil resulting in a larger spectral contribution of the soil’s initial P (~ 800 mg P/kg of crystalline calcium phosphate mineral species) for this sample rather than rapid TPP hydrolysis.

### Long-term field speciation and fate of TPP

**Fig. 3:**
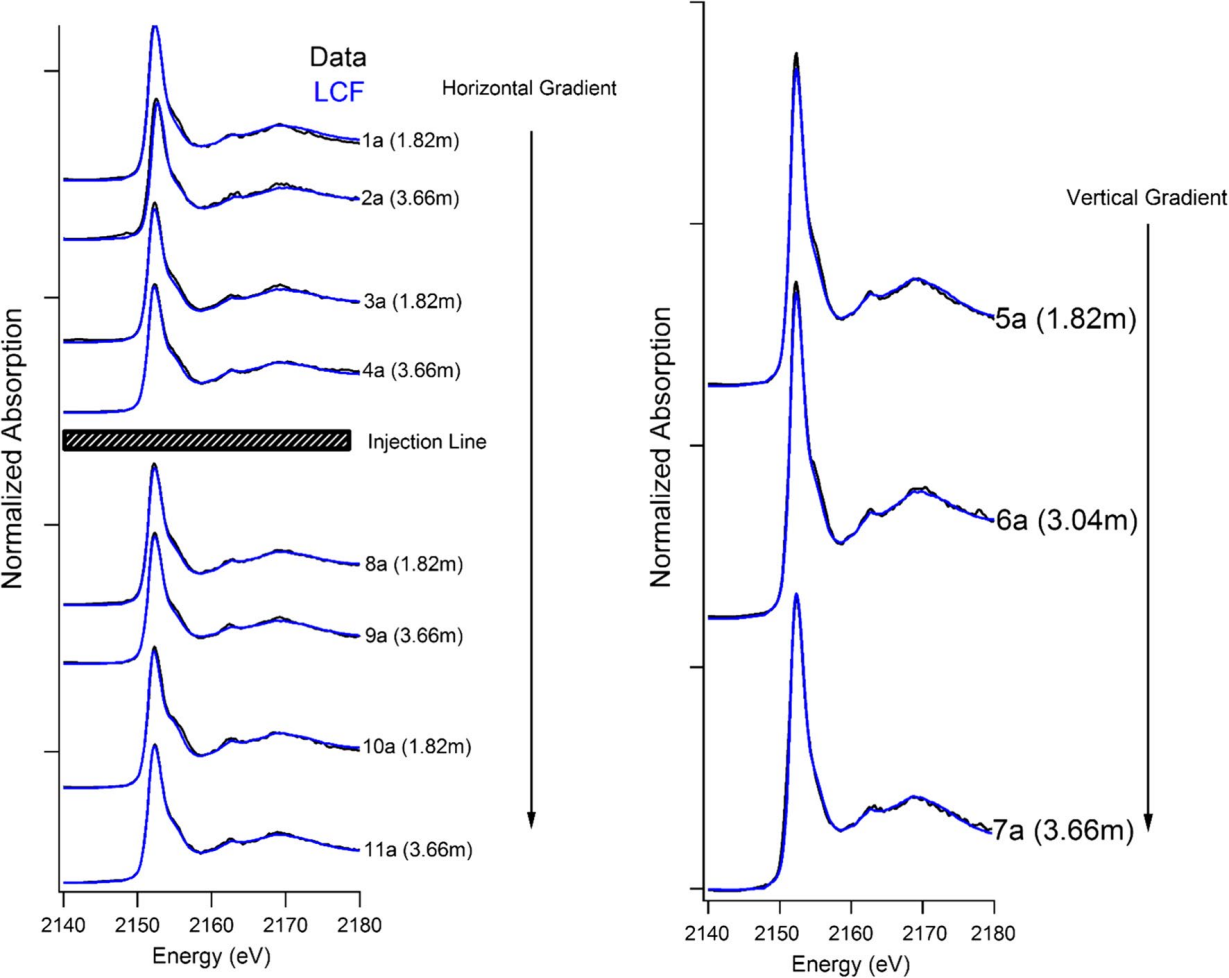
Phosphorus XANES and linear combination model fits for the horizontal and vertical hydrological gradient from the amendment injection line sampled 1 year after the first TPP application

**Fig. 4:**
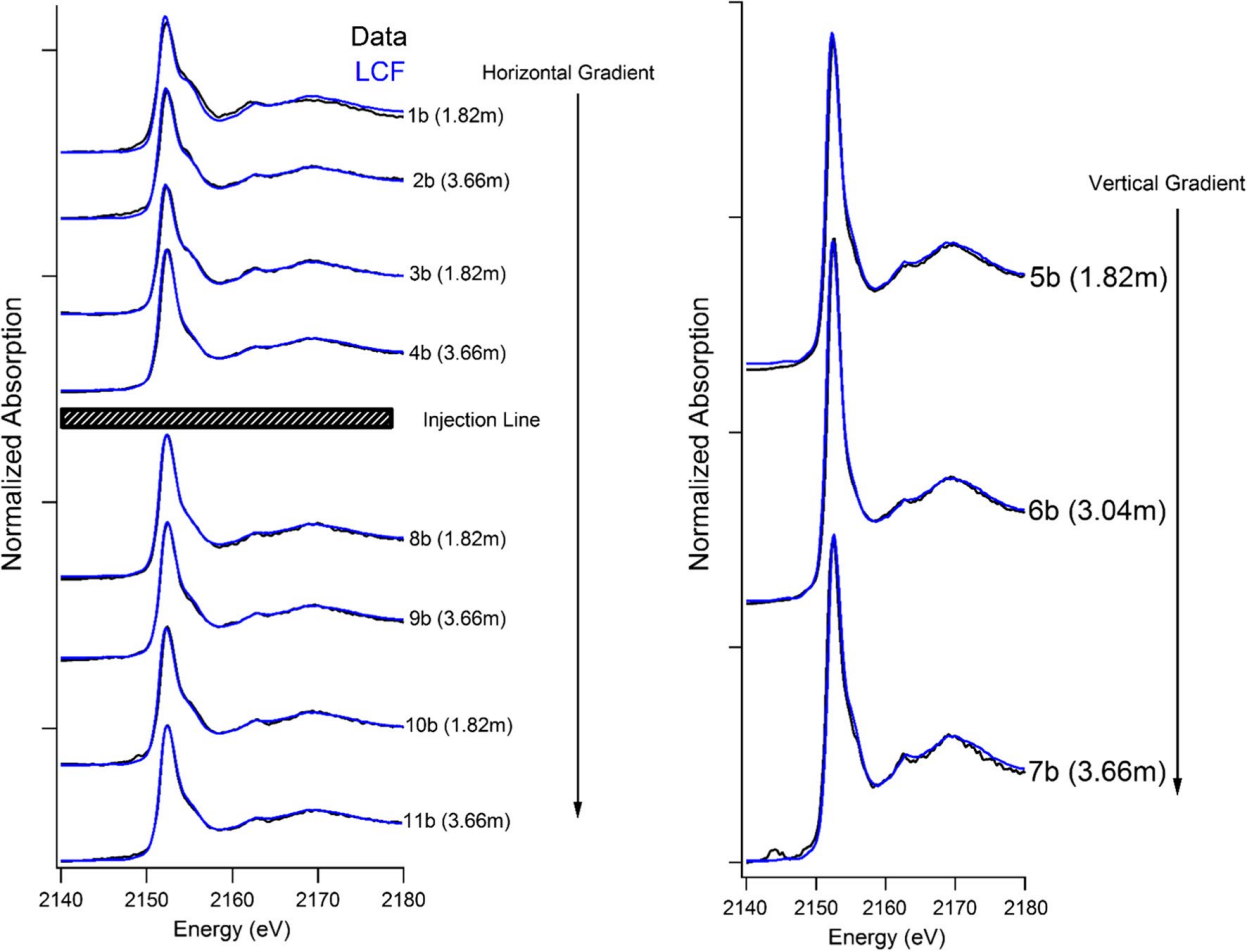
Phosphorus XANES and linear combination model fits for the horizontal and vertical hydrological gradient from the amendment injection line sampled 1 year after the second TPP application

Based upon the short-term laboratory results, we hypothesized that TPP adsorption will affect both TPP mobility and chemical fate in soils. The application of TPP to a P-limited field site will help determine the extent of TPP distribution/filtration and provide an indication of how long TPP can remain adsorbed in a natural system without hydrolysis and precipitation reactions occurring. Phosphorus XANES and LC model fits from the first TPP amendment application are displayed in Fig. 3. The results of the LCF analysis, including all soil geochemical information can be found in Table 1. The slight pre-edge feature in the “2a and 7b” XANES spectra (Figs. 3, 4), likely arises from scattering peaks from diffracting minerals that were not able to be fully normalized out in the lowest concentration samples, and is not the result of Fe phosphate mineral formation.

**Table 1:** Bulk soil chemical analysis and XAS linear combination fitting results for the first application of TPP

Label	Depth (m)	Soil^a^ pH	Total elemental concentrations^b^				Ca:Mg ratio	Labile^c^ extractable P (mg P/kg soil)	LCF analysis results^d,e^		Reduced chi^2^
			Ca (mg/kg soil)	Mg (mg/kg soil)	P (mg/kg soil)	Fe (mg/kg soil)			Adsorbed P (%)	Ca-P species (%)^f^	
1a	1.82	7.7	23,800	14,900	950	24,500	1.6:1	10	38	70	0.00141
2a	3.66	7.7	36,000	16,000	910	26,500	2.25:1	10	33	70	0.00131
3a	1.82	7.8	25,400	16,900	880	26,700	1.5:1	23	48	56	0.00310
4a	3.66	7.5	32,400	21,800	1050	19,000	1.5:1	21	48	59	0.00127
5a	1.82	7.7	28,000	15,400	880	24,500	1.8:1	5	46	58	0.00142
6a	3.04	7.7	30,300	18,000	940	29,000	1.7:1	25	42	65	0.00110
7a	3.66	7.6	10,900	9100	1080	8100	1.2:1	75	45	52	0.00127
8a	1.82	7.7	28,300	18,600	1010	28,300	1.5:1	15	42	65	0.00123
9a	3.66	7.8	28,000	17,900	950	28,000	1.6:1	15	44	58	0.00149
10a	1.82	7.7	25,700	13,400	915	25,700	1.7:1	10	24	80	0.00219
11a	3.66	7.8	28,900	17,300	950	28,900	1.6:1	20	40	62	0.00054

^a^Soil pH accurate to ± 0.1

^b^Via total XRF elemental analysis, concentrations are accurate to ± 10%

^c^Combination of H_2_O and NaHCO_3_ extractable P

^d^Eo Shift constrained to zero

^e^% Relative contribution to XAS signal; models are unconstrained and not equal to 100%

^f^Relative sum of the contribution from calcium phosphate mineral species

The low concentration TPP amendment did not increase soil P concentrations. Elemental analysis revealed (Table 1) that P concentrations are similar both directly adjacent and below the amendment injection line. Notably, there was no increase in total P along the vertical gradient closest to the injection system, which would have been expected simply based upon proximity. Labile extractable P concentrations are low relative to both total P concentrations and percentage of adsorbed P throughout all soils. As the adsorbed P fraction of the LCF models is most likely due to adsorbed TPP, this suggests that adsorbed TPP is not readily extractable or desorbed by either H_2_O or Na-bicarbonate. Similarly to the ortho-P treatment of Fig. 2, the high concentrations of Ca and relative abundance of carbonate minerals (Additional file 1: Figure S2) favour the formation of a Ca-P surface precipitate if the adsorbed P fraction was an adsorbed ortho-P molecule.

The soils closest to the amendment injection line had the highest fraction of adsorbed P. This was expected since the vertical gradient soils were in closest proximity to the amendment injection line. Based upon the widespread distribution of adsorbed P, despite the soils being high in clay, the amendment is likely traveling through preferential flow paths from the injection point to the sand lens at 3.66 m before proceeding through the sand lens. The adsorbed P fraction of the up-gradient soils provides evidence that nutrient amendment was also being forced to these locations. The best explanation for this is that the amendment solution was mounding during this initial nutrient application resulting in saturating the infiltration capacity of the soils and driving nutrient solution to up-gradient positions. The 1.82 m down-gradient soil had the lowest fraction of adsorbed P; this is likely due to a lack of amendment flow to this area of the site.

The second amendment application consisted of a more concentrated TPP solution with a smaller water volume than the first application. Phosphorus speciation results from 1 year after the second concentrated TPP application are presented in Fig. 4 (XANES spectra) and Table 2 (LCF results and geochemical information). With the increase in TPP concentration, only one soil position experienced an increase in total P, this soil was located directly adjacent to the injection system. The concentration increased from ~ 800 to ~ 3000 mg P/kg soil. Soils further away from the injection system have P concentrations largely consistent with soils from the first TPP application. Nonetheless, labile extractable P was higher after the second application, typically ~ 80 mg P/kg versus–15–20 mg P/kg. This fraction increased site-wide even though total P was largely unchanged. One explanation for this increase could be the hydrolysis of adsorbed TPP from the previous TPP application. This ortho-P could have either remained in an adsorbed form or precipitated as a soluble Ca-P species. Either species may be susceptible to desorption or dissolution by the extraction used to measure labile P.

**Table 2:** Bulk soil chemical analysis and XAS linear combination fitting results for the second application of TPP

Label	Depth (m)	Soil^a^ pH	Total Elemental Concentrations^b^				Ca:Mg ratio	Labile^c^ extractable P (mg/kg soil)	LCF analysis results^d,e^		Reduced chi^2^
			Ca (mg/kg soil)	Mg (mg/kg soil)	P (mg/kg soil)	Fe (mg/kg soil)			Adsorbed P (%)	Ca-P species (%)^f^	
1b	1.82	7.8	22,800	15,200	820	24,700	1.5:1	74	10	96	0.00743
2b	3.66	7.8	29,900	17,300	870	27,300	1.7:1	79	24	76	0.00192
3b	1.82	7.8	24,800	14,100	790	25,000	1.8:1	71	18	81	0.00074
4b	3.66	7.4	23,900	15,900	880	29,600	1.5:1	60	41	61	0.00104
5b	1.82	7.9	22,200	13,800	750	25,200	1.6:1	85	43	57	0.00117
6b	3.04	7.9	1340	3140	3230	16,700	0.4:1	85	63	40	0.00139
7b	3.66	7.6	49,700	29,400	780	29,400	3.2:1	82	32	70	0.00140
8b	1.82	7.8	25,300	15,300	880	26,700	1.7:1	19	31	71	0.00130
9b	3.66	7.8	26,300	17,000	920	28,100	1.5:1	27	36	63	0.00155
10b	1.82	7.8	33,000	15,100	960	26,900	2.2:1	24	42	61	0.00059
11b	3.66	7.5	18,100	14,400	1000	39,200	1.2:1	62	31	70	0.00100

^a^Soil pH accurate to ± 0.1

^b^Via total XRF elemental analysis, concentrations are accurate to ± 10%

^c^Combination of H_2_O and NaHCO_3_ extractable P

^d^Eo Shift constrained to zero

^e^% Relative contribution to XAS signal; models are unconstrained and not equal to 100%

^f^Relative sum of the contribution from calcium phosphate mineral species

Soils in closest proximity to the injection line had the highest relative fractions of adsorbed P. However, TPP amendment movement appears to have been limited and did not reach up-gradient soils. This is expected, as the lower water volume was unlikely to fully saturate the study area and thus would not force amendment to up-gradient positions. The small relative fraction of adsorbed P at the 1.82 m up-gradient sample is likely either residual adsorbed P from the first amendment application. The increase in adsorbed P down gradient indicates TPP can be both mobile and reactive with soil minerals. Although TPP adsorption to soil minerals reduces its expected mobility in soils, there is evidence of TPP distribution throughout the studied area as noted by increases to the relative fraction of adsorbed P.

### Effectiveness of TPP as a P amendment in calcareous soils

The adsorption and persistence of TPP between application and sampling (~ 1 year) in a calcareous soil system is an important finding. The persistence of TPP and adsorbed P in this soil environment indicates the biotic hydrolysis of TPP may be limited. While phosphatase was not directly measured in this study, potential reasons phosphatase activity could be low include: (1) reduced microbial populations as a result of PHC toxicity, (2) lack of root exudates in subsurface soils due to a history of paved surface cover, and (3) even if present in soils, some research indicates adsorbed TPP may not be readily susceptible to phosphatase catalyzed hydrolysis [16, 17].

Tripolyphosphate application increases adsorbed P and appears to be stable in this soil environment for a full year between application and sampling. In the absence of enzyme catalyzed hydrolysis of TPP, the abiotic hydrolysis of TPP in solution and soils is expected to be slow or non-existent specifically at the low temperatures consistent with this site (< 5 °C) [3, 18]. The alkaline nature of these soils further reduce the abiotic hydrolysis rates, as TPP hydrolysis is significantly faster in acidic conditions [3, 7, 18]. However, even though the hydrolysis rate is expected to be slow, there is still evidence that hydrolysis is occurring: there is an increase in labile extractable ortho-P between sampling points and there is a reduction in adsorbed P of the up-gradient soil after the second soil core sampling. High Ca concentrations and adsorption to mineral surfaces may both catalyze TPP hydrolysis and may be responsible for the hydrolysis occurring in these typically unfavorable hydrolysis conditions [7, 18].

Tripolyphosphate is capable of strongly adsorbing to minerals either in a flat or terminal configuration [8, 10], neither form of adsorbed TPP appears to be readily desorbed from soil mineral surfaces based upon the labile extraction results of this study. This was exemplified by the 2.43 m soil having the highest P concentration (~ 3000 mg P/kg soil), highest fraction of adsorbed P, but similar labile P concentrations to the surrounding soils. While adsorbed TPP may not be readily desorbed, a key finding is it does not form Ca-P mineral phases until after hydrolysis; the formation of Ca-P minerals has been shown to significantly reduce microbial P bioavailability [29]. It is expected that adsorbed TPP would be readily available to microbial communities as they would likely contain the phosphatase enzymes capable of hydrolyzing and cleaving P from linear poly-P [29, 40]. However, while research suggests that adsorbed ortho-P is bioavailable to microbes, there is no direct evidence to date that indicates whether microbial populations are capable of scavenging adsorbed TPP from mineral surfaces. Further study is required to determine whether adsorbed TPP is bioavailable. However, adsorbed ortho-P has been shown to be a preferred species for increasing potential soil P bioavailability, as it is an accessible species for microbial uptake [29].

**Fig. 5:**
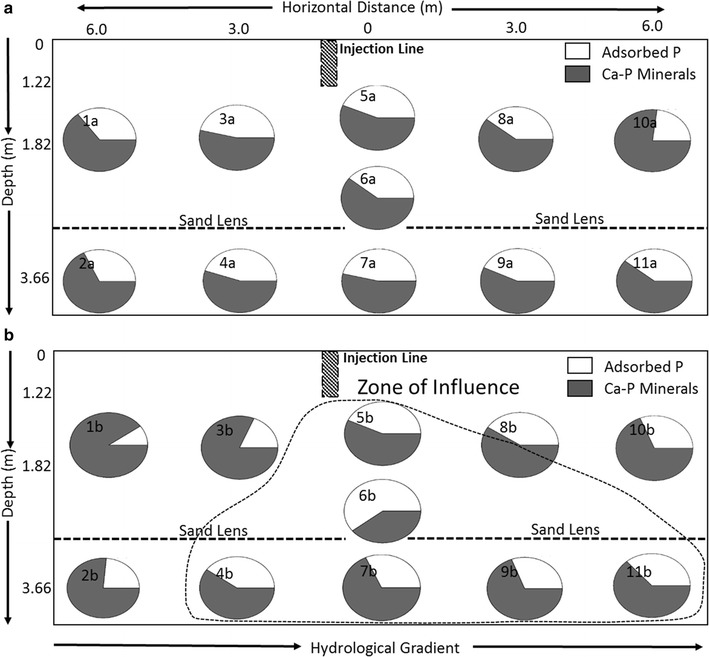
Phosphorus speciation as a 2-diminsional representation of the study site by depth and the hydrological flow of ground water after the 1st (**a**) and 2nd (**b**) applications of TPP. Indicated in panel B is the extent of the predicted zone of influence of the TPP amendment based upon the LCF results

The distribution of adsorbed P at this study site appears to be dependent on water volume/site saturation as illustrated in Fig. 5. However, both the highest relative fraction of adsorbed P and the highest total P concentration resulted from the concentrated TPP application, although with a lower zone of influence than the first application. It was expected that the low loading of TPP would be less mobile in soils, with most TPP rapidly adsorbing to mineral surfaces. In contrast, higher loadings of TPP were expected to result in the highest relative fraction of adsorbed P and elevated total P concentrations site wide. As once the adsorption sites of a mineral surface have been saturated, remaining dissolved TPP should be free to move with groundwater flow resulting in TPP distribution. Increasing total P concentrations through TPP application may be restricted by the overall adsorption capacity of the mineral surfaces; soils may require multiple applications to allow TPP time to hydrolyze. The high sorption affinity of TPP on mineral surfaces reduce the risk of TPP moving offsite or into untargeted areas causing unintended P-related ecosystem damage.

## Conclusions

Liquid TPP amendments have proven to be an effective P source for facilitating and maintaining adsorbed P on soil mineral surfaces in Ca rich environments. This research has shown that TPP will rapidly (> 48 h) adsorb on soil surfaces and persist primarily as adsorbed P in a calcareous soil environment. While these results are consistent with a number of short-term laboratory complexation studies of TPP adsorption and hydrolysis on metal oxides, this is one of the first studies to measure TPP complexation onto soils. However, the bioavailability of adsorbed TPP is unclear and warrants further study to determine whether microbes are capable of utilizing this P source from mineral surfaces. Tripolyphosphate adsorption presents a challenge to distributing TPP throughout a subsurface soil profile due to impeding TPP transport. It was found that the movement of dilute concentrations of TPP is dependent on ground water flow and appears to rely upon large water volumes to transport amendment throughout the site. When concentrated TPP applications with decreased water volume were utilized, they resulted in higher relative fractions of adsorbed P and localized total P increases, but decreased site coverage of adsorbed P. Applying high concentrations of TPP with large volumes of water may be a more effective strategy for increasing the concentration and distribution of adsorbed P throughout this PHC contaminated site.

## Additional file

**Additional file 1: Figure S1.** Comprehensive library of phosphorus XANES reference standards used during linear combination fitting. **Figure S2.** X-ray diffraction of the vertical gradient borehole (US05) located directly adjacent to the amendment injection line. Dashed lines indicate the major carbonate phase’s calcite (C) and dolomite (D) (see Additional file: Table S1 for mineral phases and Rietveld refinement).

## Abbreviations

ACP: amorphous calcium phosphate
Ca-P: calcium phosphate minerals
CLS: Canadian Light Source Synchrotron
LCF: linear combination fitting
Ortho-P: orthophosphate
PHC: petroleum hydrocarbon contamination
Poly-P: polyphosphate
Pyro-P: pyrophosphate
TPP: tripolyphosphate
XAS: X-ray absorption spectroscopy
XANES: X-ray absorption near edge structure
XRD: X-ray diffraction
XRF: X-ray fluorescence
