# Supplementary material for: Chemical speciation and fate of tripolyphosphate after application to a calcareous soil
Source: Geochem Trans. 2018 Jan 8;19:1. doi: 10.1186/s12932-017-0046-z (PMC5758486; doi:10.1186/s12932-017-0046-z)
Supplement: Supplementary file 1 — Additional file 1: Figure S1. Comprehensive library of phosphorus XANES reference standards used during linear combination fitting. Figure S2. X-ray diffraction of the vertical gradient borehole (US05) located directly adjacent to the amendment injection line. Dashed lines indicate the major carbonate phase’s calcite (C) and dolomite (D) (see Additional file: Table S1 for mineral phases and Rietveld refinement). [file 12932_2017_46_MOESM1_ESM.docx]

**Additional information**

Phosphorus XANES standards, and X-ray Diffraction results

**
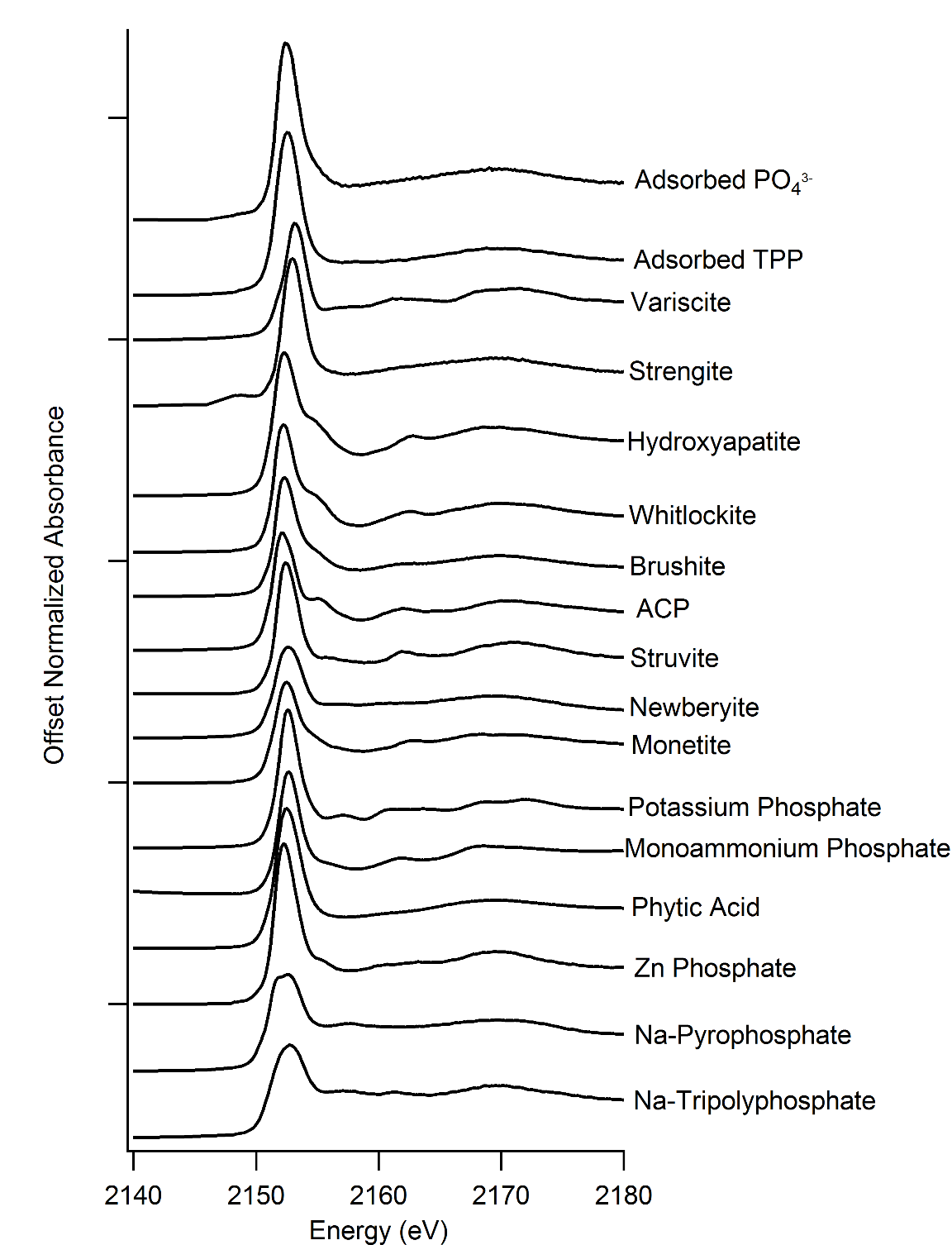
**

**Fig. S. 1. Comprehensive library of phosphorus XANES reference standards used during linear combination fitting.**

**Phosphorus XANES reference compounds**

The adsorbed ortho-P standard was synthesized by reacting K_2_HPO_4_ with goethite for 48 hrs under pH 6.5 conditions in a batch reactor. The maximum loading, given 100% adsorption, was calculated to be 10000 mg P kg^-1^ of goethite. The goethite filtrate was triple washed with P-free background electrolyte to remove any entrained ortho-P. The adsorbed TPP reference standard was synthesized by reacting Na-TPP in a goethite suspension with a 0.01 M CaCl_2_ background electrolyte. The maximum loading, given 100% adsorption, was calculated to be 10000 mg P kg^-1^ goethite, but it is expected that less than 100% adsorption occurred. The suspension was then syringe filtered through a 0.45 µm filter, triple washed with P-free background electrolyte, and then freeze dried for XAS measurement. These P loadings were chosen to coincide with potentially calculated maximum soil P concentrations, as well as to ensure high quality XANES spectra. Phosphorus adsorbed on Al oxide mineral surfaces was not considered during LCF analysis due being nearly spectrally identical to adsorbed P on Fe oxide mineral surfaces and the inability to distinguish between the adsorbed phases within a soil matrix (1).

The calcium phosphate mineral standards (hydroxyapatite, whitlockite, brushite, amorphous calcium phosphate, struvite, monetite, and newberyite) were synthesized by Hilger (2017), please see for synthesis methods and mineral verification. The remaining mineral and organic P reference compounds were purchased as concentrated reagent grade compounds from Sigma-Aldrich and Fischer Scientific. The P mineral reagent grade standard compounds were verified via X-ray diffraction.

**
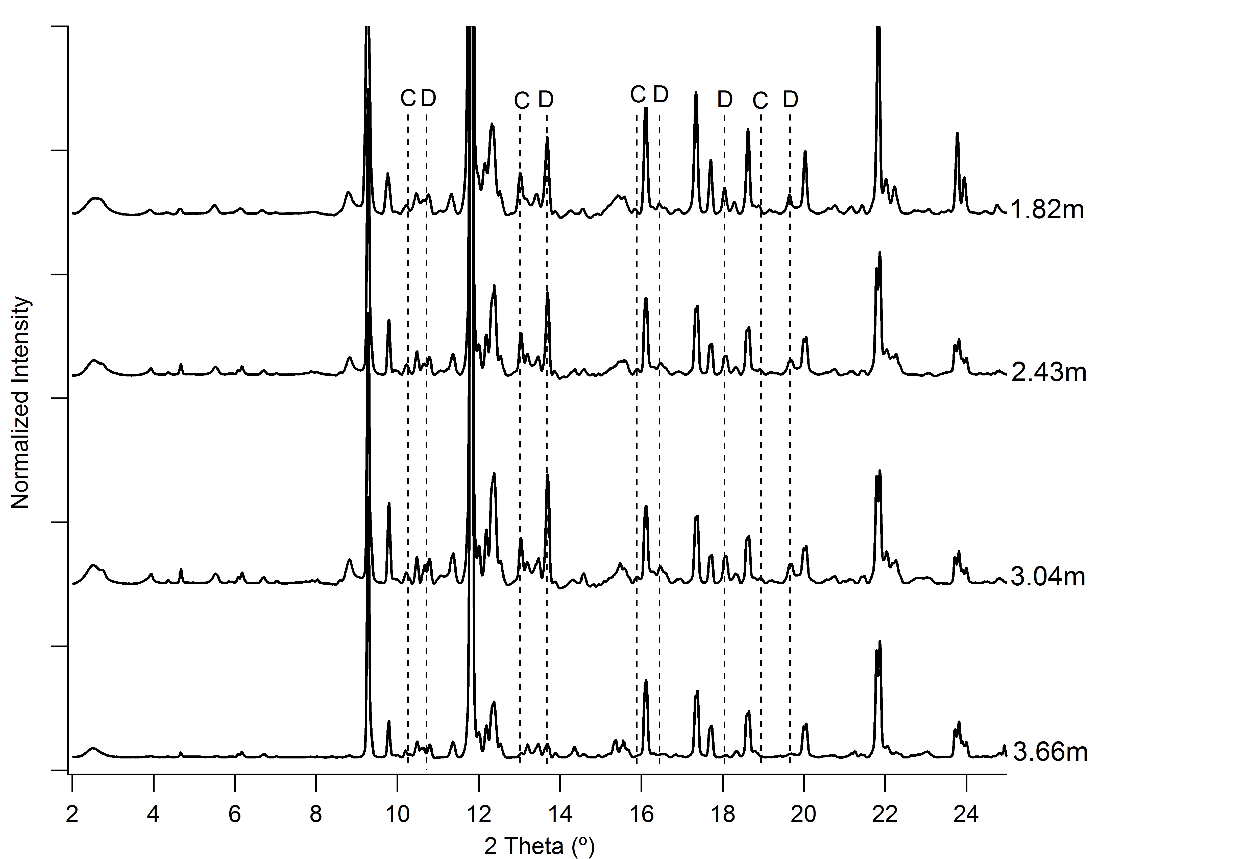
**

| Sample (Depth ) | Quartz | Albite | | **Dolomite** | **Calcite** | Mica | Microcline | Clinochlore |
| --- | --- | --- | --- | --- | --- | --- | --- | --- |
|  |  | | Relative Abundance (Wt. %) | | | | | |
| 1.82m | 61 | 11 | | **5** | **2** | 6 | 8 | 4 |
| 2.43m | 40 | 11 | | **4** | **1** | 33 | 4 | 5 |
| 3.04m | 50 | 21 | | **6** | **2** | 7 | 8 | 3 |
| 3.66m | 71 | 12 | | **1** | **0.2** | 3 | 7 | 1 |

**Fig. S. 2. X-ray diffraction of the vertical gradient borehole (US05) located directly adjacent to the amendment injection line. Dashed lines indicate the major carbonate phase’s calcite (C) and dolomite (D) (see Supplemental Information Table S.1 for mineral phases and Rietveld refinement).**

**Table S. 1.** Rietveld refinement of the major identified mineralogical phases of the US-05 borehole located directly adjacent the TPP injection line. Highlighting the carbonate mineral components of the soil mineralogy that may place an important role in P fate.

**Soil Mineralogy**

Soil mineralogy is an important factor when studying P speciation, as mineral surfaces are important reactants in both adsorption and surface precipitation of P (2). To understand the chemical fate of TPP in soils, it is important to identify the reactive mineral surfaces and how they vary spatially across the site. Bulk XRD completed on the vertical soil core directly adjacent to the amendment injection line (Fig. S. 2 [US05 borehole], Supplemental Information), indicated that the mineralogy of the core varies minimally with depth, the differences tend to be texture-based with the major mineral phases varying only in their relative abundance. All the soils throughout the research site were found to contain significant weight percent quantities of dolomite (~5%) and calcite (~2%) which represent two mineralogical phases that can potentially interact with the applied TPP amendments. These carbonate minerals can directly sorb P and form Ca-P mineral surface precipitates with any applied or naturally present P (3,4). The groundwater across the site was found to have dissolved Ca and Mg concentrations of 200-400 and 150-350 mg L^-1^, respectively. Concentrations of this magnitude may lead to any aqueous ortho-P precipitating in the soil solution as Ca/MgP mineral phases, further reducing dissolved P.

**References**

1. Beauchemin S, Hesterberg D, Chou J, Beauchemin M, Simard RR, Sayers DE. Speciation of phosphorus in phosphorus-enriched agricultural soils using X-ray absorption near-edge structure spectroscopy and chemical fractionation. J Environ Qual. 2003;32(5):1809–19.

2. Hesterberg D. Macroscale chemical properties and X-ray absorption spectroscopy of soil phosphorus. Dev Soil Sci. Elsevier Masson SAS; 2010;34:313–56.

3. Karaca S, Gürses a., Ejder M, Açikyildiz M. Kinetic modeling of liquid-phase adsorption of phosphate on dolomite. J Colloid Interface Sci. 2004;277(2):257–63.

4. Tunesi S, Poggi V, Gessa C, Scienze UCI, Agroindustriali T, Agraria C. Phosphate adsorption and precipitation in calcareous soils : the role of calcium ions in solution and carbonate minerals. Nutr Cycl Agroecosystems. 1999;53:219–27.
